# Soil to Cytoplasm: The Mycorrhizal Phosphorus Express and Its Regulatory Steps

**DOI:** 10.3390/jof12070473

**Published:** 2026-06-26

**Authors:** Rizwan Ali Ansari, Kobilov Ergash Egamberdievich, Batirov Xidir Fayziyevich, Tukhtaev Mustafa Kurbonovich, Belyalova Leylya Enverovna, Madjidova Tanzila Raximovna, Rustamova Rano Babakulovna

**Affiliations:** Department of Ecology and Life Safety, Faculty of Geography and Ecology, Samarkand State University Named After Sharof Rashidov, Samarkand 140104, Uzbekistan

**Keywords:** phosphate transporter genes, cellular localization, symbiosis, plant health, computational modelling

## Abstract

Mycorrhizal symbiosis shows the most widespread evolutionarily ancient strategy by which higher plants overcome phosphorus limitations in soil. The acquisition of phosphorus by terrestrial plants is fundamentally constrained by low soil mobility and strong chemical fixation. Arbuscular mycorrhizal symbiosis overcomes these limitations through a regulated transport system that regulates phosphate movement across distinct control points such as soil extraction, internal hyphal translocation, and the host–fungal exchange interface. This review provides a mechanistic synthesis of each checkpoint, and evaluates how up- and downregulatory genes, specific phosphate transporter families, and environmental conditions collectively determine symbiotic efficiency. We integrate recent advances in molecular genetics, computational modelling, and artificial intelligence to resolve the spatial and temporal dynamics of phosphate movement from soil to plant cell. The analysis reveals that the phosphorus journey from soil to cell represents a chain of tightly regulated transport events through the multiple checkpoints. Each checkpoint dictates the regulation of phosphorus, and helps in nutrient exchange judiciously. By mapping these regulatory stops, the review establishes a comprehensive framework for the optimization of biological phosphorus acquisition. The review also determines when and why proper symbiosis fails and ways to overcome such critical feature. The synthesis identifies critical regulatory nodes and outlines targeted strategies to enhance biological phosphorus acquisition, optimize crop nutrient-use efficiency, and reduce reliance on mineral fertilizers in sustainable agricultural systems.

## 1. Introduction

Phosphorus is one of the macronutrients that is very important for the growth of all plants. Since phosphorus is in such limited supply, it is the limiting macronutrient for all plants. Phosphorus is taken up by plants as inorganic ortho-phosphate (Pi) and this form of phosphorus is not very soluble or very mobile, and it becomes immobilized in most soils very quickly [1]. The concentration of Pi in the soil solution does not reach higher than 10 µM, whereas the cytoplasmic concentration of Pi in plants is at millimolar (mM) levels [2,3]. This large difference in concentration between the soil and the plant creates a major bioenergetic cost for the plant to take up phosphorus directly through the roots [4,5]. Approximately 80% of all land plants form a mutualist relationship with the arbuscular mycorrhizal (AM) fungi within the subphylum Glomeromycotina [6]. The AM fungal hyphae reach beyond the root phosphate depletion zone and deliver Pi to the host roots through specialized structures called arbuscules [7]. This end result is that there is now a second phosphorous acquisition method through symbiotic association with AM fungi that operates similarly and often in parallel with the first phosphorous acquisition method through direct root uptake [8].

**Figure 1:**
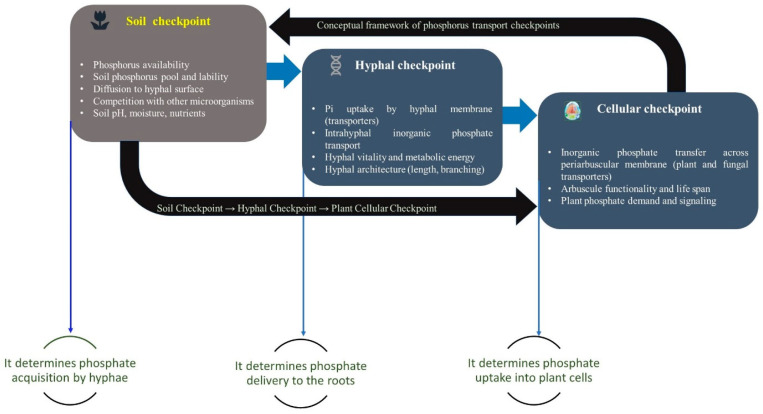
A conceptual framework that describes three main regulators governing mycorrhiza-mediated Pi uptake from soil to the plant cell.

The transport of phosphorus from the soil to a plant’s cytoplasm is accomplished through a series of highly regulated transport events and a number of membranes, and is accomplished with the help of transporter proteins that mediate each of the individual steps involved. For instance, in the extraradical mycelium, AM fungi employ high-affinity Pi transporters such as the PHO84 family to absorb Pi from the soil solution against a concentration gradient [9,10]. Once inside the fungal hyphae, Pi undergoes rapid conversion into polyphosphate (polyP), a linear polymer that serves as both a reservoir and a transport vehicle. PolyP is translocated along the hyphae to the intraradical mycelium, where it is hydrolyzed back to Pi before export into the symbiotic interface [10]. At the arbuscule, the fungal Pi exporters discharge Pi into the periarbuscular space. Thereafter, plant-encoded mycorrhiza-inducible phosphate transporters (predominantly members of the PHT1 family) import Pi into the cortical cell cytoplasm [5]. Overall, this cascade of transport reactions constitutes that there may be metaphorically said as the “mycorrhizal phosphorus express” a dedicated, highly efficient system which helps to channel soil phosphorus into the plant cell (Figure 1).

However, efficient phosphate transport in the mycorrhizal pathway needs precise and specific regulation to maintain nutrient homeostasis and the well-functioning of symbiosis. The plant exerts stringent control over symbiotic phosphate delivery through a multilayered regulatory network that adjusts the activity, abundance and localization of transport components according to the current phosphorus status of plants [11]. A central regulatory hub is the PHR-SPX signalling module. Under low-Pi conditions, PHOSPHATE STARVATION RESPONSE (PHR) transcription factors such as AtPHR1 in *Arabidopsis* and OsPHR2 in rice dimerize and bind to PHR1 binding site (P1BS) motifs in the promoters of phosphate starvation-induced genes, which include those encoding mycorrhiza-inducible Pi transporters [12]. Under high-Pi conditions, SPX domain proteins (named after SYG1, PHO81, and XPR1) inhibit PHR dimerization and DNA binding, thereby repressing the expression of Pi-responsive genes [13]. Inositol pyrophosphates, particularly InsP8, serve as intracellular Pi status signals that modulate SPX-PHR interactions, and enable fine-tuned control over the symbiotic phosphate acquisition programme [13]. Beyond this core module, phytohormones (strigolactones, auxins, cytokinins, and gibberellins), microRNAs (miR399 and miR156), and CLAVATA3/EMBRYO-REGULATING (CLE) peptide signals integrate phosphorus availability with AM symbiosis development at multiple checkpoints [14]. Despite considerable progress, major gaps persist in the mechanistic understanding of the mycorrhizal phosphorus express and its regulatory stops. The identity of the fungal Pi exporter that delivers Pi into the periarbuscular space remains elusive [7]. The thermodynamic constraints governing nutrient exchange at the symbiotic interface, including the coupling of Pi export to cation fluxes and proton gradients, are only beginning to be resolved [15]. The post-translational regulation of mycorrhiza-inducible plant Pi transporters (such as trafficking, phosphorylation, and degradation) has been largely unexplored as compared to the well-characterized regulation of direct-pathway transporters [11]. Furthermore, the crosstalk between phosphorus status and other nutrient signalling pathways in the regulation of symbiotic function requires deeper investigation [12].

In this review, therefore, we follow the trajectory of phosphorus from soil to cytoplasm, and examine each transport step as a checkpoint along the mycorrhizal express. We first describe the molecular nature of fungal Pi uptake and polyP metabolism in the extraradical mycelium. We then examine the mechanisms of Pi translocation and export at the fungal–plant interface with the emphasis on recently proposed candidate exporters and transport mechanisms. Next, we discuss the mycorrhiza-inducible PHT1 transporters (plant side reception system), their structural features, substrate specificity, and subcellular localization at the periarbuscular membrane (PAM). We devote subsequent sections to the regulatory architecture that tunes the express to the plant’s phosphorus demands, and cover the PHR-SPX master switch, small RNA regulators, and emerging roles of post-translational modifications. Finally, we outline key knowledge gaps and propose future directions for the enhancement of phosphorus acquisition and utilization through AM fungi to assist the sustainable agriculture system.

## 2. Checkpoint 0

### 2.1. Pre-Colonization Signalling and Host Decision

This checkpoint represents the earliest gate in the mycorrhizal phosphorus express. Before fungal hyphae ever contact the root, the plant must broadcast a “welcome signal” (strigolactones; SLs) into the rhizosphere [10]. Whether this signal is sent or suppressed depends entirely on the plant’s internal Pi status. Plant–fungus communication unfolds as a highly regulated bidirectional molecular dialogue at this pre-symbiotic interface, where active communication through signalling ensures signal compatibility, establishment of fungal colonization, host-recognition, timing of the germination of the spores and the extent of fungal root colonization [16,17,18].

#### 2.1.1. SL Biosynthesis Under Pi Deficiency

Under Pi limited conditions, plant roots synthesize and exude SLs, which are carotenoid-derived terpenoid lactones. The biosynthesis of SLs requires a conserved pathway. For example, DWARF27 (a plastid-localized isomerase) converts all-trans-β-carotene to 9-cis-β-carotene, which is sequentially cleaved by carotenoid cleavage dioxygenases MORE AXILLARY GROWTH3 (MAX3) and MAX4, and then oxidized by the cytochrome P450 MAX1 [19]. Pi deficiency strongly upregulates D27, MAX3, and MAX4 transcript levels, massively boosting SL production. Once exuded, SLs act as a potent chemoattractant for AM fungi; they stimulate spore germination and hyphal branching at concentrations as low as 10^−13^ M [20]. Mechanistically, SLs rapidly increase mitochondrial density and metabolic activity in fungal cells, which trigger a proliferative response that primes the fungus for root contact [20,21]. In essence, under Pi deficiency, plants emit signalling molecules that recruit AM fungi to the root surface. Moreover, SL exudation is not a phosphate-only response but a compressed output of a wide regulatory network spanning nitrogen, sulfur, drought, heat, light, pathogens, and neighbouring plant roots, which are filtered through shared transcriptional gatekeepers into a single chemical signal in the soil [22,23,24,25].

#### 2.1.2. PHR-SPX Control of SL Production

The decision to produce SLs is hardwired into the canonical plant phosphate starvation response (PSR) system. The central transcriptional activator is PHOSPHATE STARVATION RESPONSE1 (PHR1; in rice, OsPHR2), an MYB transcription factor that binds to P1BS cis-elements in the promoters of Pi-starvation-induced genes such as D27, MAX3, and MAX4 [12]. Under Pi-sufficient conditions, PHR1 is kept inactive by SPX domain-containing proteins (SPX1/SPX2/SPX4). These SPX proteins act as Pi-dependent molecular switches. For instance, when cellular Pi is high, they directly bind PHR1 (or PHR2), block its dimerization and DNA-binding capacity, and repress SL biosynthetic genes [26,27]. When Pi drops, inositol pyrophosphate levels fall, SPX proteins lose their affinity for PHR1 and are degraded, unleashing PHR1 to activate SL production and thus symbiosis takes place [28]. This PHR-SPX module, therefore, acts as a Pi status-to-symbiosis transducer. It ensures that the host invests in fungal recruitment only when internal Pi reserves are truly deficient. SPX proteins function as the sentinel that prevents wasteful symbiosis initiation under adequate Pi supply [12].

#### 2.1.3. High-Shoot Pi Suppresses AM Colonization

The host decision is not purely local, it also involves long-distance communication. Split-root experiments in pea have shown that Pi supply to one part of the root system systemically suppresses AM colonization including hyphopodia formation and SL exudation. Other roots also exhibit suppression of SL exudation and mycorrhization [29,30], which indicates that a shoot-transported systemic signal overrides local Pi deficiency signals. This indicates that a shoot-derived systemic signal overrides local Pi deficiency signals. When shoot Pi concentration is high and reflects sufficient plant-wide Pi status, the shoot sends a suppressive cue (likely via phloem-mobile microRNAs [31], hormones such as cytokinin [32], or Pi itself [33]) that downregulates SL biosynthesis genes in roots systemically [14]. This multilayered regulation prevents the plant from investing carbon into symbiosis when shoot Pi reserves are already adequate.

## 3. Checkpoint I

### 3.1. The External Mycelium: Mining Soil Phosphorus

The network of extraradical mycelium occupies a much larger area of soil volume, as it continues to grow beyond the root depletion zone. By doing this, extraradical mycelium is able to utilize the phosphorus fractions that are bound to minerals and organic molecules in the soil.

#### 3.1.1. Extraradical Hyphal Networking and Exploration of Pi

AM fungi utilize extraradical hyphae, having extended beyond the Pi depletion zone, which is generally found in close proximity (1–2 mm) to the root [34]. Extraradical hyphae have access to areas of soil that are not reached directly by roots. AM fungi are able to gather Pi present in soil patches that are far away from the plant and return it to the host plant [35,36]. The degree to which different species of AM fungi fill their extraradical hyphal network varies; some species produce longer hyphae for the purpose of extending further into the soil while other species may depend on their associated hyphosphere microbiomes to mobilize organic phosphorus in the vicinity [37,38]. The biofilms containing phosphate-solubilizing bacteria (PSB) are associated with the hyphal surface, allowing for the mineralization of organic phosphorus in more distant soil patches [39]. Additionally, the extraradical hyphae secrete glomalin-related soil proteins and necromass and contribute to increased carbon sequestration in soils from the removal of the rhizosphere [40,41]. The mechanisms by which Pi is taken up from the soil into extraradical hyphae by fungal Pi transporters are fairly well known, yet the molecular basis of Pi efflux from a fungus into a plant at the site of symbiosis remains not fully understood [7]. Coordination between the systemic signalling pathways via the PHR-SPX regulators enables whole-plant coordination of both Pi-starvation responses and mycorrhizal colonization [12].

#### 3.1.2. Accessing Recalcitrant P Pools: Phosphatases and Organic Phosphorus Mineralization

Accessing recalcitrant phosphorus pools requires coordinated biochemical, microbial, and molecular mechanisms that go far beyond the mere spatial exploration of soil by roots or mycorrhizal networks. Under phosphorus limitation, root systems and fungal partners exude organic acids, notably citrate, malate and oxalate, which mobilize soil phosphorus through the chelation of Al^3+^, Fe^3+^ and Ca^2+^ ions [42,43,44]. The PSBs complement fungal processes through the mobilization pathways of Pi [45,46]. Such PSBs enhance phosphorus availability via the phosphatase-mediated hydrolysis of organic phosphorus, acidification of the rhizosphere, and the chelation of metal-bound phosphorus using low-molecular-weight organic acids, including gluconic and ketogluconic acids [46,47]. The solubilisation of Pi is primarily driven by organic acid and proton release, whereas enzymatic hydrolysis assists in the mineralisation of organic phosphorus [48].

The enzymatic component of this system relies on an enzyme (phosphatases) that catalyzes the hydrolysis of organic phosphorus compounds, leading to enhanced mineralisation and phosphorus release [49,50,51]. However, AM fungi possess intrinsic phosphatase production capacity, and they frequently recruit bacterial groups to supply these enzymes, especially alkaline phosphatases, which establishes a functional interdependence within the mycorrhizosphere [47,52]. This interaction is further supported by the fungal exudation of carbon substrates such as fructose, which considerably stimulate bacterial phosphatase production and enhance cooperative phosphorus mobilization [47]. Established PSBs, such as *Pseudomonas*, *Rhizobium* and *Bacillus*, contribute through organic acid secretion, rhizosphere acidification and the disruption of mineral phosphate complexes, which enhance phosphorus solubility and limit re-fixation, and improve micronutrient availability [53,54]. Collectively, these interlinked fungal–bacterial interactions help in accessing recalcitrant phosphorus and their judicious mobilization.

#### 3.1.3. The Hyphosphere Microbiome

**Figure 2:**
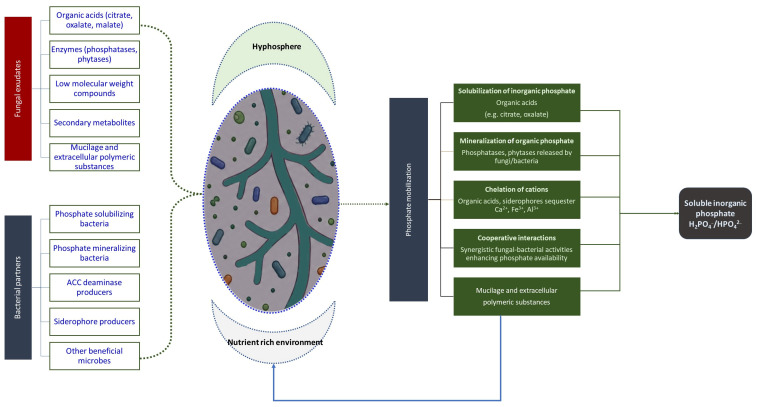
An overview of hyphosphere interactions including fungi and PSBs, and the possible mechanisms of Pi mobilization.

The hyphosphere constitutes a distinct soil microenvironment wherein AM fungi appropriately recruit a specialized microbial consortium that compensates the inherent fungal limitations in phosphorus mobilization (Table 1; Figure 2). This core hyphosphere community exhibits marked taxonomic conservation across divergent edaphic conditions, and rapidly establishes within fourteen days of initial hyphal soil contact. Extraradical hyphae consistently enrich members of the Betaproteobacteriales, Myxococcales, Fibrobacterales, Cytophagales, Chloroflexales and Cellvibrionales, which advocates a conserved recruitment mechanism [55,56]. While Glomeromycota possess the genomic capacity to synthesize phosphatases, they often depend upon bacterial associates to mineralize organic complex phosphate (phytate) and mobilize Pi [57,58]. Broader hyphosphere communities, such as *Azotobacter*, *Microbacterium*, *Burkholderia*, *Enterobacter*, *Flavobacterium*, *Erwinia*, *Rhizobium and Serratia*, further sustain Pi solubilisation and organic phosphorus turnover, as well as corroborate host plant nutrition and rhizobial functioning [59,60]. The ecological architecture of this microbial network reveals notable plasticity in response to agricultural management. Network analyses indicate that intercropping systems promote significantly stronger positive associations between AM fungi and hyphosphere bacteria compared with monocultures, thereby shifting community structure towards a more synergistic state that optimizes phosphorus solubilisation [61]. We are in opinion that such reports establish the hyphosphere as a highly regulated micro-niche where carbon-for-phosphorus exchanges drive coordinated microbial assembly, functional specialization and nutrient mobilization across different environmental and agronomic contexts.

#### 3.1.4. The Carbon: Phosphorus Trade-Off in the Hyphosphere

The carbon economy of mycorrhizal phosphorus acquisition is governed by differential plant investment strategies that reflect the accessibility of phosphorus sources. Plants accessing phosphorus bound to secondary minerals such as goethite exhibit substantially higher carbon costs per unit phosphorus acquired, with increased transfer of both structural and energy storage lipids to fungal partners relative to readily available phosphorus sources [69]. The carbon-to-phosphorus ratio thus functions as an indicator of the metabolic cost associated with mobilizing distinct phosphorus pools, evidencing a regulated adjustment of carbon allocation in response to phosphorus bioavailability [69,70]. At the plant level, carbon allocation is accompanied by systemic metabolic reprogramming that prioritizes symbiotic function over growth. This reconfiguration involves hormonal adjustments which include the upregulation of SLs and downregulation of gibberellins, and the redistribution of carbon from structural growth towards energy storage and symbiotic maintenance [71,72]. Such regulation reflects a resource trade-off in which phosphorus acquisition efficiency is prioritized under conditions of limitation.

## 4. Checkpoint II

### 4.1. The Fungal Conduit: Translocation, Transformation, and Delivery

The AM fungi operates as a sophisticated biological tool, orchestrating the capture, chemical transformation and long-distance transport of phosphorus from soil to plant cells. This fungal conduit represents a critical control point in the mycorrhizal phosphorus supply chain, where molecular machinery, cellular logistics, and metabolic transformations converge to enable efficient nutrient delivery [10]. Extraradical hyphae acquire phosphorus from the soil and transport it through the fungal network predominantly in the form of polyphosphate. At the arbuscular interface, phosphorus undergoes conversion and transfer to root cortical cells, which establishes an efficient pathway between soil phosphorus reserves and plant nutrition [73].

#### 4.1.1. High-Affinity Pi Uptake at External Hyphae

AM fungi use specialized proton-coupled symporters from the PHT1 family to help obtain nutrients at the soil–hyphal junction, where Pi levels are normally low (micromolar). Extensive genome comparisons in *Rhizophagus irregularis* identified several high-affinity Pi transporters with evolutionary links to the plant PHT1 family, including the GintPT and its orthologs [74,75,76]. Extensive spatial separation between these transporters on the extraradical mycelium has been documented, with different isotopes found at the tips of searching hyphae or in older hyphal sections. Kinetic studies of these transporters yielded K_m_ values of between 2 and 15 μM consistently, demonstrating that the affinity is sufficient to remove Pi from extremely low concentrations in severely depleted soil solutions [77]. Transcription of the genes encoding these transporters is quickly repressed by elevated levels of Pi in the external environment. This is accomplished by post-translationally tagging (endocytosis) and then degrading of the transporter proteins [78]. Structural studies of fungal PHT1 proteins have primarily relied upon homology models based on yeast Pho84 and plant PHT1 protein structures, since PHT1 proteins in fungi do not readily form crystals. The homology models suggest that PHT1 proteins maintain an overall architecture characteristic of the 12-transmembrane-domain architecture known for major facilitator superfamily proteins, and that a conserved pore structure found in the third and eighth transmembrane-domain helices coordinate Pi through specific hydrogen bond interactions. SPX domain proteins in fungi regulate intracellular Pi by serving as molecular receptors for inositol pyrophosphates that modulate the transporter’s localization and activity at the root–hyphal interface [79]. Therefore, the Pi uptake system in fungi exists as a regulated process with high-affinity transport and rapid feedback inhibition that allows for effective scavenging while preventing excess to metabolism overload.

#### 4.1.2. Polyphosphate (PolyP): The Fungal Currency for P Translocation

As Pi enters the fungal cytosol, it is rapidly converted to inorganic PolyP, an orthophosphate-based linear polymer representing the metabolic currency of the symbiosis. This process is facilitated through the incorporation of functional homologues of the VTC complex on the tonoplast of external hyphae [80]. Thus, when Pi is converted from a diffusible into an osmotically inert form of polyphosphate, a steep chemical concentration gradient is maintained across the plasma membrane, and feedback inhibition is avoided, so that phosphorus can continue to be taken up under varying environmental fluctuations [10]. In addition, PolyP serves as a buffering system, providing a means for the transient storage and regulated release of phosphorus when host demand exceeds what is available from the surrounding environment. Long-distance transport of PolyP occurs along a continuous tubular vacuolar network extending from the extraradical mycelium to the root cortex. Based on the results of transcriptomic and proteomic analyses, there is abundant evidence to suggest that the metabolism of PolyP is compartmentalized so that polyphosphatases and regulatory kinases are upregulated where polymer hydrolysis and phosphate release occur [10]. As such, PolyP is not only a means of storage, but serves as the primary logistical medium within the mycorrhizal network to supply phosphorus to the host [81].

#### 4.1.3. Intraradical Hyphal Networks: Distribution and Branching Logic

The intraradical hyphae that comprise the reticulate network in the root cortex facilitate the redistribution of phosphorus through a pattern of branching dependent on the local environment. Branching of the hyphae follows the model of resource allocation, therefore, cortical areas that are high in carbon are likely to stimulate the initiation of arbuscules, whereas areas low in phosphorus are likely to stimulate further branching to access adjacent cells. The life cycle of the AM structures is regulated and takes approximately 3–7 days for its completion [17,82,83]. The regulated breakdown of the arbuscules and the clearance of the mycorrhizal structures by the host prevents excessive build-up of fungal biomass around the host plant. Quantitative imaging demonstrates that the density of branching, the morphology of arbuscules and the turnover rate of arbuscules regulate phosphorus delivery to the host inside the fungal network, thereby establishing a self-regulated feedback mechanism that balances soil resource exploration with nutrient exchange between the host and fungal partner [84,85,86]. In our opinion, nutrient transport and delivery in the intraradical space is facilitated through a mechanism that links the allocation of carbon by the host to the delivery of phosphorus from the fungal partner. This mechanism is cyclical in nature and serves to maintain mycelial responsiveness and facilitate an uninterrupted flow of phosphorus to the host while preventing metabolic stasis.

#### 4.1.4. Mechanisms of Pi Export at Arbuscular Interface

The arbuscule is the centrepiece of the AM symbiosis, functioning as the primary exchange interface between the plant and the fungus through the highly branched and transient filament structures of the fungus, surrounded by a plant-derived membrane system that allows for the bidirectional exchange of nutrients [84,85]. While the arbuscule is a well-studied structure, there is still a lack of understanding regarding the molecular basis for the export of Pi from the fungus into the periarbuscular space. Canonical fungal PHT transporters have not been identified, which suggests that other efflux pathways may be operating. Among the proposed alternatives are SPX domain proteins, which may control Pi efflux through conformational gating, and SYG1/XPR1 homologues, which are known to facilitate Pi export in other eukaryotic systems [10,79]. Structural predictions of these proteins indicate that they may exist as oligomeric channels with differences in charge distribution, facilitating the movement of Pi along electrochemical gradients. Additionally, there is evidence for three different, but non-mutually exclusive, mechanisms of Pi export. These include (1) direct efflux of Pi through an unidentified transporter into the periarbuscular apoplast; (2) localized hydrolysis of PolyP within the arbuscular hyphae by exopolyphosphatases to release Pi; and (3) vesicle-mediated transport of Pi or PolyP-containing vesicles that fuse with the PAM to release their contents to the outside environment [87]. The fungal partner does not have the ability to generate the essential fatty acids needed for their metabolism and relies completely on host-derived lipids that are transported across the PAM by STR/STR2 transporters, demonstrating the degree of metabolic interdependence between the two partners [88,89,90]. All the transcriptional changes observed in single-cell transcriptome analyses of cortical cells laser-capture microdissected from *Medicago truncatula* indicate that arbusculated cells are actively up-regulating genes to support PAM biogenesis and lipid remodelling, as well as expressing the mycorrhiza-specific phosphate transporter MtPT4, while the adjacent uncolonized cells remained at their basal levels of gene expression [91,92]. Collectively, the absence of confirmed fungal Pi exporters remains an unresolved mechanistic question; however, current evidence supports a model of multiple mechanisms of Pi export that include direct efflux, enzymatic hydrolysis of PolyP, and vesicle-mediated transport. After Pi translocate across the PAM, it can then enter the intraradical distribution network.

#### 4.1.5. Fungal Pi Efflux into the Symbiotic Interface

Phosphate export from the fungal partner into the periarbuscular space represents the least understood link in the mycorrhizal phosphorus supply chain [7]. Moreover, no fungal Pi efflux protein had been molecularly identified for decades [93]. Candidate transporters emerged through phylogenomic surveys of mycorrhizal fungal genomes paired with heterologous expression systems [7]. In *Rhizophagus irregularis*, SPX domain proteins and putative phosphate transporters (e.g., RiPT) likely govern Pi export, though direct efflux assays remain scarce [10]. Phosphate export must overcome a steep electrochemical gradient, and the periarbuscular apoplast carries a positive membrane potential and millimolar Pi concentrations [1]. This thermodynamic barrier suggests either a proton-coupled symport reversed in direction, a Pi/H^+^ antiport, or an ATP-binding cassette transporter [6].

Carbon supply from the plant regulates fungal Pi efflux. Imbalanced carbon-for-phosphorus exchange downregulates fungal phosphate transporter transcription [94]. The plant Pi status also feeds back on the fungus, and phosphorus-starved roots increase SL exudation, whereas phosphorus-replete roots suppress colonization and possibly Pi export [93]. Once Pi enters the interface, plant-encoded phosphate transporters (e.g., MtPT4, OsPT11) capture it with high affinity [1,95]. An open question includes whether Pi efflux occurs only at arbuscule branch tips or along the entire arbuscule surface, whether the same transporter family operates in other important mycorrhizal fungi, and what post-translational switches trigger export only when the host plant provides sufficient carbon [6,7].

## 5. Checkpoint III

### 5.1. The Symbiotic Interface: Phosphate-for-Carbon Exchange

The trade floor of symbiotic relationships between plants and most mycorrhizal fungi is known as the arbuscule where the integrated functions of the mycorrhizal and plant transporters will define how much phosphate gets moved into the plant, or how much carbon is obtained from the plant. To be reciprocal and fair, this mutualistic exchange must have defined regulation.

#### 5.1.1. Mycorrhiza-Specific PHT1 Transporters: Identification and Their Localization

Within AM symbiosis, PHT1 phosphate transporters are central to phosphate transfer from fungal partners to host plants. Specific members of the PHT1 family are induced or strongly upregulated during AM formation and localize to the PAM at the arbuscule branch domain [96,97] (Table 2). Mycorrhiza-associated PHT1 transporters are typically categorized into clade I, comprising mycorrhizal-specific members, and clade III, which encompasses mycorrhizal-inducible transporters [96]. Clade I transporters are restricted to arbusculated cells and exhibit progressive upregulation that mirrors the intensity of root colonization [96]. Two representative systems, MtPT4 from *Medicago truncatula* and OsPT11 from rice, have been extensively characterized as model AM phosphate transporters [96,98,99]. MtPT4 is highly expressed in mycorrhizal roots and mediates phosphate uptake directly from arbuscules, with localisation confined to the plasma membrane that envelops arbuscular branches [91,100,101]. The rice orthologue OsPT11 shows comparable localisation and functional behaviour, and isotope tracing has revealed that phosphate transfer via the fungal pathway is abolished in its absence [102,103]. Beyond nutrient transport, AM-inducible PHT1 transporters contribute to the regulation of arbuscule development and its lifespan [104]. GFP-tagged OsPT11 studies further illustrate the dynamic regulation of transporter localisation and expression during arbuscule development [100]. Collectively, MtPT4 and OsPT11 exemplify the functional and evolutionary specialization of AM-specific PHT1 transporters.

AM colonization consistently induces the expression of specific PHT1 phosphate transporters across diverse angiosperm, which proves a conserved molecular adaptation for symbiotic nutrient acquisition. This transcriptional response manifests across a diverse range of taxa, where mycorrhizal-inducible transporters exhibit tissue-specific localisation within colonized root tissues and enhanced activity under phosphorus-deficient conditions. For example, tomato roots strongly activate LePT3, LePT4 and LePT5 under phosphorus limitation during mycorrhizal association [105]. The conservation of this regulatory mechanism extends to woody species such as poplar, where PtPT9 and PtPT12 respond to both arbuscular and ectomycorrhizal colonization that indicate broader evolutionary framework for symbiotic phosphate transport [106]. Thus, conserved PHT1 induction marks a universal symbiotic strategy. Fungal phosphate delivery becomes host capability. This molecular lever, precisely regulated, also reshapes crop phosphorus efficiency.

Moreover, PHT1 phosphate transporters are specifically localized to the PAM surrounding arbuscule branches, which is the principal interface of active nutrient exchange during AM symbiosis. These high-affinity, proton-coupled transporters are transcriptionally induced or strongly upregulated during AM formation and are essential for unidirectional phosphate transfer from the fungal partner to the plant host [97,107]. The phosphate confinement to the PAM reflects a highly specialized spatial organization of phosphate uptake machinery at the symbiotic interface. Experimental evidence from fluorescent protein fusion studies provides direct confirmation of this localisation. Transgenic expression of MtPT4 and OsPT11 fused to green fluorescent protein under native promoters advocates exclusive accumulation of both transporters within the PAM domain surrounding arbuscule branches engaged in nutrient exchange [96]. Consistently, MtPT4 from *Medicago truncatula* is expressed only in arbusculated cells, and is localized to PAM which surround the arbuscular structures [100]. Collectively, these findings establish that symbiosis-specific PHT1 transporters are precisely targeted to the PAM, where their spatial confinement supports a highly specialized and directional system for phosphate transfer across the plant–fungal interface.

#### 5.1.2. SPX-PHR Regulatory Hub

SPX (SYG1/Pho81/XPR1) domain proteins constitute the primary phosphate-sensing module that regulates PHR transcription factor activity through direct protein–protein interactions [108,109]. Although SPX domains do not directly bind Pi, they perceive phosphate status indirectly via inositol polyphosphate signalling molecules that reflect cellular phosphate availability [110]. Under phosphate-sufficient conditions, SPX1 and SPX2 interact with PHR1 in a phosphate-dependent manner and act as competitive inhibitors, and prevent PHR1 binding to P1BS promoter and suppress phosphate starvation responses [26,111]. In AM symbiosis, SPX proteins perform dual regulatory functions across developmental phases. Under phosphate starvation, SPX1 and SPX3 promote the expression of SLs biosynthetic genes such as DWARF27, and enhance SL exudation that stimulates fungal attraction and activation in the rhizosphere [112]. Following symbiosis establishment, these proteins become spatially restricted to arbuscule-containing cells, where they contribute to regulated arbuscule turnover and the maintenance of symbiotic balance [112]. The SPX-PHR module functions as a central regulatory switch integrating direct phosphate uptake with mycorrhiza-mediated acquisition pathways. Under phosphate-replete conditions, SPX proteins suppress both PHR2-dependent symbiotic gene expression and excessive phosphate uptake, thereby limiting unnecessary investment in mycorrhizal colonization [12,113]. In tomato, SlSPX1 physically interacts with multiple PHR proteins (SlPHR1, SlPHR4, SlPHR10, SlPHR11 and SlPHR12), coordinating phosphate transport and AM symbiosis in response to phosphate availability [114]. Loss of SlSPX1 function leads to enhanced mycorrhizal colonization even under phosphate-sufficient conditions which confirm their role in avoiding inappropriate symbiotic engagement [114].

In addition, PHR1 (PHOSPHATE STARVATION RESPONSE 1) and its homologues constitute the central transcriptional regulatory hub governing Pi homeostasis in plants [115,116]. These MYB-CC (MYB coiled-coil) transcription factors function as dimers that bind P1BS (PHR1 Binding Site) motifs with the consensus sequence GnATATnC in promoters of Pi-responsive genes [117,118]. Collectively, PHR1, PHL1 and PHL2 collectively regulate a significant portion of phosphate starvation-responsive transcriptome which makes them master regulators of cellular phosphate acclimation [117,119]. Overall, PHR1 and related MYB-CC transcription factors function as master regulators integrating phosphate homeostasis and AM symbiosis through P1BS-mediated gene regulation, under stringent control by SPX proteins and inositol polyphosphate signalling to prevent inappropriate activation under phosphate sufficiency.

#### 5.1.3. Post-Transcriptional and Post-Translational Regulation: PTMs, Trafficking, and miRNA

Post-translational regulation provides a further control layer over phosphate transporter function. It modulates the activity, localisation, and stability of PHT1 proteins. This prevents phosphate overaccumulation under phosphate-sufficient conditions. It also enables rapid adjustment to environmental fluctuations [116]. The regulation of protein phosphorylation is critical to control the trafficking of the phosphate transporter PHT1. PHT1 transporters are phosphorylated by casein kinase 2 (CK2) at the CK2α3/β3 complex under nutrient-rich or phosphate-rich conditions. Once phosphorylated, PHT1 transporters remain sequestered in the endoplasmic reticulum and do not traffic to the plasma membrane; therefore, this mechanism limits the capacity of the plant to uptake phosphate when there is sufficient phosphate in the plant tissues. CK2-mediated retention of the PHT1 transporters is a critical checkpoint during mycorrhizal symbiosis to avoid toxin accumulation in aerial plant tissues because of toxic levels of phosphate [120].

In addition to phosphorylation, microRNAs (miRNAs) represent a significant mechanism of post-transcriptional regulation associated with phosphate homeostasis. miRNAs exert fine control over the level of phosphate transported and signalled by means of sequence-specific degradation of the target mRNAs. In the plant shoot tissues, the conserved miRNAs are upregulated under conditions of phosphate deprivation, and they are transported from the shoot to the roots through the phloem. They target and induce degradation of the transcripts encoding major regulatory proteins [12,113,121]. The overall response of the miRNA-coordinated degradation of the transcripts results in the suppression of the ubiquitin-mediated degradation pathways that normally reduce the abundance of phosphate transporters in plants under phosphate-replete conditions. Therefore, phosphate starvation simultaneously increases transporter expression and stabilizes transporter proteins in plants. In mycorrhizal systems, miR399 is critical to maintaining the homeostasis of phosphate in colonized roots through its regulatory input into major facilitator genes. The high level of upregulation and control of miR399 over transporters in conjunction with its regulatory effect illustrates its importance for mediating the cellular balance of phosphate during mycorrhizal symbiosis [122]. Therefore, miRNA networks provide a means of regulating the integration of direct uptake systems with symbiotic phosphate acquisition.

Fungi also exhibit additional layers of control over phosphate homeostasis, particularly via epigenetics (i.e., DNA methylation, especially 6-methyladenine (6 mA)). Regulation of genes involved in phosphate transport and signalling through DNA methylation in AM fungi has been proven [123]. Phosphate scarcity causes a switch in signal transduction pathways employed in plants, the PKA pathway is activated when phosphate is limited, and the MAPK and TOR pathways are inhibited when phosphate is limited. These signalling pathways show methylation-associated controls; therefore, it appears that there are epigenetic regulatory networks controlling phosphate-responsive signalling networks [123]. Collectively, these contributions allow for the entire fungal metabolic response to fluctuate with the availability of phosphate. Therefore, phosphate regulation through AM symbiosis appears to represent an integrated, multilayered approach with miRNA-mediated post-transcriptional control and common signalling networks working together to regulate phosphate uptake, the stability of transporters, and symbiotic balance between the plant and fungal partners.

#### 5.1.4. Carbon-for-Phosphate Exchange and Biological Market Theory

The SWEET family of transporters carries sugars as the main form of carbon, and facilitates them to periarbuscular space [124,125,126]. After fungi use these six carbon sugars, sugars for fungi are transported into the cell by monosaccharide transporters, which operate with varying specificity towards different sugars [107,126]. In parallel, AM fungi take up phosphorus from the soil matrix via high-affinity transporters (typically H^+^/P transporters) located on the extraradical mycelial network [29]. At the symbiotic interface, fungal polyphosphatases depolymerise these stores to liberate free phosphate for plant uptake [126,127]. Phosphate efflux into host root cells occurs at the arbuscular periphery and necessitates coordinated transporter activity, which may incorporate SPX domain-containing proteins alongside proton-coupled symporters [126,127,128]. The bidirectional exchange relies upon complementary molecular machinery that harnesses electrochemical proton gradients. Both symbionts employ proton pumps coupled to H^+^/sugar and H^+^/phosphate transporters to drive nutrient flux with high specificity [125].

In addition, mycorrhizal symbiosis can be interpreted through biological market theory, in which phosphate-for-carbon exchange functions as a cooperative market-like system governed by reciprocal valuation and differential reward [125]. A central feature of this system is bidirectional partner choice. Host plants detect and preferentially reward fungal partners that provide greater access to phosphate through increased allocation of photosynthate, whereas fungal partners enhance nutrient transfer to roots supplying greater quantities of carbohydrates [125,129]. This reciprocal preferential allocation has been directly observed, with plants that allocate more carbon to fungi delivering greater phosphate returns and fungi preferentially directing phosphate towards hosts with stronger carbon supply [130,131]. Collectively, these findings support a conceptually integrated view of mycorrhizal symbiosis as a biologically regulated marketplace in which both plants and fungi exercise partner choice, reward efficient exchange, and reduce allocation to less beneficial partners.

**Table 2:** Phosphate transporters in mycorrhizal symbiosis across plant species functional diversity and regulatory mechanisms.

PHT1 Transporter Gene	Plant Species	AM Fungal Partner	Expression Pattern During AM Colonization	Cellular Localisation	Functional Evidence/Phenotype	Reference
EgPT8	*Eucalyptus grandis*	*Rhizophagus irregularis*	Activated specifically by AM fungus	Localized in root cortical cells containing arbuscules	Knockdown does not affect growth or phosphorus concentration, but suppresses mature arbuscule size; cannot complement defects in mutants	[132]
SmPHT1;6	*Salvia miltiorrhiza*	*Rhizophagus irregularis*	Strongly expressed in roots colonized by AM fungi	Subcellular localization prediction revealed that all the SmPHT1 proteins were located on the plasma membrane	Induced expression under AM fungi colonization; significant impact on growth and phosphorus accumulation under different P conditions	[133]
TaPT29-6A	*Triticum aestivum*	*Funneliformis mosseae*	Highly upregulated in AM-colonized roots	Localized in plasma membrane	Required for AM fungal colonization; TaPT29-6A silencing reduces arbuscule formation and increases susceptibility to biotrophic, hemi-biotrophic and necrotrophic pathogens	[134]
AtPT1, AtPT2, AtPT4, AtPT9, and AtPT11	*Acer truncatum*	*Rhizophagus irregularis*	AM colonization upregulated AtPT4 and AtPT11, with AtPT11 having a specific induction pattern for mycorrhizal phosphorus acquisition.	Localized in Plasma membrane	AtPT4 linked to phosphorus uptake. AtPT1 is involved in phosphorus remobilization within plant tissues, AtPT2 in phosphorus transport and remobilization (suppressed by AM colonization), and AtPT9 in phosphorus uptake and transport efficiency under high-phosphorus conditions.	[135]
LePT1–8 LePT3, LePT4, LePT5; LePT1, LePT2, LePT6, LePT7	*Solanum lycopersicum*	*Glomus intraradices*	Under low-phosphorus conditions, LePT3, LePT4 and LePT5 strongly induced in AM-colonized roots, but not under high-phosphorus conditions; expression restricted to cells containing fungal structures. In contrast, LePT1, LePT2, LePT6 and LePT7 significantly downregulated in mycorrhizal roots under low Pi. LePT8 transcript was found undetectable in all tissues.	Distinct root cortical cells harbouring AM fungal structures (reported for promoter-driven GUS expression of LePT3 and LePT5)	Promoter analysis showing 1250 bp LePT3 and 471 bp LePT5 promoter fragments containing MYCS and P1BS cis-elements are sufficient to drive GUS expression specifically in mycorrhizal roots.	[105]
HvPHT1;11, HvPHT1;11.2, HvPHT1;12, HvPHT1;13.1/13.2	*Hordeum vulgare*	*Glomus* sp.	AM colonization of roots specifically upregulated HvPHT1;11, HvPHT1;11.2, HvPHT1;12 and HvPHT1;13.1/13.2	All proteins except HvPHT1;4 lack any signal peptide sequence and are predicted to be mostly plasma membrane-localized. The preferred localization of HvPHT1;4 appears to be in the chloroplast.	AM inoculation improved phosphorus use efficiency and seedling growth; transcript accumulation confirmed AM-specific induction of subfamily II PHT1 genes in roots; the study reveals beneficial role of AM fungi in barley growth promotion under P-related stress	[136]
AsPT1, AsPT4	*Astragalus sinicus*	*Gigaspora margarita* (BEG34) or *Glomus intraradices* (BEG141).	Triggered in Pi-starved mycorrhizal roots; both AsPT1 and AsPT4 are induced under AM + Pi-starvation conditions	Both AsPT1 and AsPT4 localized in plasma membrane	AsPT1 overexpression increased mycorrhization and arbuscule abundance; AsPT1 knockdown caused degenerating/dead arbuscules (similar to AsPT4 silencing); AsPT4 required for symbiotic Pi uptake; AsPT1 proposed as novel symbiotic transporter essential for AM development	[137]
PHO1-1, PHO1-2, PHT1-3, PHT1-7	*Trifolium repens*	*Funneliformis mosseae*	Upregulated in roots of AM fungi-inoculated plants under Al stress compared with control plants; associated with enhanced mycorrhizal colonization	Not reported	AMF inoculation reduced Al accumulation, increased root phosphorus content, enhanced plant biomass, and improved Al stress tolerance; upregulation of PHT1-7 associated with improved phosphorus uptake	[138]
SlPT3, SlPT4, SlPT5 (within the SlPHT1 family; total PHT genes identified = 23)	*Solanum lycopersicum*	*Funneliformis mosseae*	Upregulated under AM colonization at all Pi conditions tested (0, 25, and 200 µM Pi); expression significantly increased following AM inoculation	Not reported	AM colonization was strongest under low Pi (25 µM); AM fungi significantly affected P and N accumulation and root morphological plasticity; indicates that AM inoculation mainly alters PHT1 family expression involved in inorganic phosphate transport	[103]
SlPT3 (PHOSPHATE TRANSPORTER 1 (PHT 1) gene family member)	*Solanum lycopersicum*	*Rhizophagus irregularis*	Strongly induced under simultaneous Pi and Zn deficiency during AM symbiosis; mycorrhiza-activated expression; responsive to combined Pi–Zn stress conditions	Not mentioned	Required for Pi transport and arbuscule development under Zn deficiency; knockdown caused reduced Fe accumulation and arbuscule degeneration; heterologous yeast expression complemented Δpho84 Pi uptake defect and influenced Fe homeostasis (fet3fet4 mutant)	[139]

## 6. Checkpoint IV

### 6.1. Systemic Distribution, Loading and Long-Distance Transport of Pi

Pi, which is acquired by the mycorrhizal network from the soil through the roots of plants, must move from the root to the leaves or to the fruits produced through photosynthesis to support the growth of the whole plant and improve overall yield. Mycorrhizal-acquired Pi is systematically distributed from root to shoot and reproductive organs through PHO1-mediated xylem loading and phloem-driven remobilisation, fuelling photosynthesis, nucleic acid synthesis, and ATP production at every stage of plant development [1].

#### 6.1.1. PHO1 Family in Pi Loading into Xylem

Once Pi reaches the root stele via symplastic or apoplastic routes, its commitment to long-distance transport hinges on xylem loading, a process mediated by the PHO1 family. The PHO1 was identified through map-based cloning of the pho1 mutant, which is selectively defective in transferring Pi from root cells to the xylem vessels yet retains normal Pi uptake capacity [140]. PHO1 encodes a protein with a hydrophilic N-terminus and six C-terminal transmembrane domains, exhibiting no homology to canonical Pi:H^+^ symporters which place it in a novel transporter class [140]. The *Arabidopsis* genome harbours 11 PHO1 homologs, though only PHO1 and PHO1; H1 can functionally rescue xylem loading in the pho1 mutant; and both are expressed in the stellar cells of roots and the lower hypocotyl [141]. However, PHR-1 regulates the expression of PHO1; H1, while PHO1 expression is not influenced by both PHR-1 and phosphite [141]. In the mycorrhizal context, the PHO1 proteins likely handle the Pi that has been unloaded from the arbuscule–cortical cell interface (via PHT1 family transporters) and subsequently channelled through the symplast toward the stele [8]. Thus, PHO1 acts as the gatekeeper that converts root-acquired Pi into a xylem-borne systemic resource.

#### 6.1.2. Vacuolar Pi Storage and Retrieval: SPX-MFS Proteins

The vacuole constitutes the largest intracellular Pi reservoir, buffering cytoplasmic Pi concentrations against external fluctuations and metabolic demands. The long-sought vacuolar Pi transporters were identified as SPX-MFS proteins which is also designated PHT5 or VPT (Vacuolar Phosphate Transporter), belonging to a subclass of SPX domain-containing proteins fused to a major facilitator superfamily transmembrane domain [142]. In *Arabidopsis*, loss of PHT5;1 reduces vacuolar Pi accumulation and lowers the vacuolar-to-cytoplasmic Pi ratio by approximately 40%, while overexpression drives massive Pi sequestration into vacuoles and alters the expression of Pi-starvation-responsive genes [142]. Heterologous expression of rice OsSPX-MFS1 in yeast demonstrates that these transporters mediate Pi influx into the vacuolar lumen, establishing the vacuole as a Pi sink during nutrient sufficiency [142]. Conversely, other SPX-MFS members operate in the reverse direction. For example, OsSPX-MFS3 functions as a vacuolar Pi efflux transporter, releasing stored Pi back into the cytosol under low-Pi conditions and thereby maintaining cytoplasmic Pi homeostasis in rice [143]. OsSPX-MFS1, predominantly expressed in leaves, plays a key role in the regulation of Pi homeostasis in photosynthetic tissues [144]. Collectively, the SPX-MFS proteins equip plants with a dynamic vacuolar buffer that can absorb excess Pi when supply is high and remobilize it when external Pi is scarce.

#### 6.1.3. Shoot-to-Root Systemic Signalling and Shoot Pi Status Feedback

Long-distance Pi allocation is not a unidirectional pipeline but is governed by a sophisticated shoot-to-root signalling loop that tailors root Pi acquisition to shoot demand. Systemic signalling was established through split-root experiments and grafting studies showing that Pi-starved shoots can communicate deficiency signals to roots independently of local Pi concentrations [103,145,146]. A central systemic messenger is the microRNA miR399, which is induced under Pi deficiency in shoots, loaded into the phloem, and transported to roots where it downregulates PHO2, which is an E2 ubiquitin conjugase that negatively regulates Pi uptake and translocation [147]. The phloem-mobile signals are perceived in root vascular tissues, where they adjust the expression of PHO1 and PHT1 transporters, effectively closing the feedback loop [121,148]. Notably, recent work has shown that the PHR-SPX system also orchestrates AM symbiosis, connecting Pi status to the extent of mycorrhizal colonization [121]. Thus, the systemic feedback from shoot Pi status coordinates the entire phosphorus express from mycorrhizal uptake to vacuolar buffering to xylem loading, ensuring that investment in Pi acquisition matches whole-plant demand.

## 7. When and Why the Symbiosis Fails

The severe abiotic stress imposes strong constraints on mycorrhizal symbiosis, with outcomes determined largely by stress intensity. Under severe stress, the mycorrhizal functioning through different structural degradation (arbuscule formation, nutrient transport) is influenced [149].

### 7.1. High-Phosphorus Fertilization: The Ultimate Check Point Breaker

AM symbiosis is strongly regulated by soil phosphorus status, with elevated phosphorus that suppress fungal colonization through an energy-conserving negative feedback mechanism. Because AM fungi consume a substantial proportion of host photosynthates, plants reduce investment in this costly partnership when phosphorus is sufficiently available through direct uptake pathways [150]. Consequently, prolonged inorganic fertilizer application lowers plant dependence on AM fungi, reduces carbohydrate allocation to fungal partners, and negatively affects fungal diversity, spore development, and hyphal production [47,151]. Moreover, under phosphorus sufficiency, SPX proteins act as negative regulators by repressing PHR-mediated transcription and inhibiting mycorrhizal infection [113,146]. In tomato, SlSPX1 directly interacts with multiple SlPHR proteins to suppress AM symbiosis marker genes, which illustrates the coordinated regulation of direct and mycorrhizal phosphorus uptake routes [114,152]. The response follows a clear dose-dependent threshold pattern. Maximum root colonization and spore production occur at 0.005–0.02 mg P g-1 soil, beyond which effectiveness declines [153]; however, negative effects can begin at fertilization rates as low as 16 μg P g-1 soil in prairie grasses, with significant inverse correlations between phosphorus concentration and colonization [154]. Overall, the evidence shows that mycorrhizal suppression depends on soil nutrient status and crop types.

### 7.2. Host Control Arbuscule Lifespan and Senescence

AM symbiosis operates under strict host surveillance. The plant does not passively accept fungal colonization but actively regulates the lifespan of arbuscules [14,155]. When the symbiosis becomes unprofitable for the host, the plant triggers arbuscule degeneration, leading to symbiosis failure. A central mechanism involves the Pi transporter PT4, located at the PAM. In *Medicago truncatula*, disruption of MtPT4 abolishes symbiotic Pi transport and causes premature arbuscule degeneration (PAD), wherein arbuscules collapse soon after formation and the symbiosis cannot be sustained [156]. This reveals that the plant monitors the delivery of its nutritional reward; when Pi transfer fails, the host terminates the arbuscule. The PAD in pt4 mutants is suppressed under nitrogen deprivation, which suggests integration of host information about both phosphorus and nitrogen status before deciding to senesce the fungal structure [156]. The suppression of arbuscule degeneration in nitrogen-starved conditions also requires the ammonium transporter AMT2;3, which further reinforces the idea that the plant’s nitrogen status also modulates arbuscule lifespan [157]. In addition, plant Pi status itself exerts powerful control over arbuscule development. At high-Pi availability, plants inhibit new arbuscule formation [158,159]. The PHR-SPX signalling module, a canonical Pi-starvation response system, governs this regulation. Under Pi sufficiency, SPX proteins inhibit the transcription factor PHR, which suppress AM symbiosis genes, while under Pi deficiency, SPX proteins are degraded and PHR activates both Pi-starvation responses and AM symbiosis programmes [12,160]. The PHR-SPX system thus ensures that the plant invests in symbiosis only when external Pi is limiting [28]. Thus, arbuscule senescence is not a passive consequence of fungal ageing but is actively orchestrated by the host as a regulatory mechanism to terminate a non-functional symbiosis.

### 7.3. Environmental Stresses and Symbiosis Dysfunction

The most well-characterized environmental trigger of symbiosis failure is high phosphorus availability in the soil. A high supply of phosphates inhibits the colonization of AM fungi via a plant-mediated regulatory mechanism [161,162]. The plant’s level of phosphates then provides information about the regulation of the genes necessary for symbiotic phosphate transport and arbuscule formation [93]. If the plant is supplied with high levels of phosphates, it reduces the amount of carbon allocated to the fungal partner, thereby limiting the ability to develop a symbiotic relationship, since an exchange of nutrients between partners is a prerequisite for establishing a functional relationship [5]. The plant shifts from mycorrhizal dependence on plant roots to uptake directly from the root system; thus, the symbiotic phosphate transport machinery is inactive [7]. Other abiotic stresses such as salinity, drought, heavy metal contamination, and extreme temperatures directly impair the survival, development, and metabolic activity of AM fungi. These stresses reduce hyphal growth, spore germination, and the capacity to colonize host roots [163]. Drought and salinity interfere with the osmotic balance of fungal cells, while heavy metals induce considerable oxidative damage to the membrane integrity and nutrient transport capacity [164]. Soil compaction and waterlogging reduce oxygen availability, to which AM fungi are particularly sensitive [165]. Millar and Bennett [166] proposed that persistent abiotic stress decreases AM fungal abundance and diversity, and thus eliminate the fungal partner pool required for functional symbiosis.

The core of the symbiosis is the reciprocal exchange of plant-derived carbon for fungal-delivered phosphate. Environmental stresses disrupt this exchange either by reducing plant carbon supply (e.g., under low light or drought) or by impairing the fungal enzymatic machinery for phosphate uptake and translocation [163]. When the cost of carbon investment exceeds the nutritional benefit derived from the fungal partner, the plant may actively restrict colonization, which leads to a breakdown of the mutualistic relationship. The efficiency of the external mycelium in phosphate uptake depends on soil conditions, and any stress factor that alters soil physico-chemical properties, such as pH extremes or nutrient stoichiometry shifts, impairs the fungal network’s ability to deliver phosphate to the root [164,165].

## 8. Systems-Level Integration of Omics and Computational Modelling

### 8.1. Omics-Enabled Network Biology of Mycorrhizal Phosphorus Transport

Recent advances in high-throughput omics technologies have enabled us to understand the molecular machinery underlying mycorrhizal phosphorus transport. Transcriptomic profiling of AM symbiotic fungi using RNA-seq has provided unprecedented insights into the coordination of gene expression of the plant host and fungal symbionts during AM symbiosis. Handa et al. [167] conducted simultaneous RNA-seq transcriptional profiling of *Lotus japonicus* and *Rhizophagus irregularis*, providing evidence for tightly regulated and coordinated gene expression programmes that are involved with nutrient exchange at the interface of symbiosis. In addition to these transcriptomic studies, genome-wide examinations of AM fungi reveal conserved nutrient signalling pathways including cAMP-PKA, SNF1, TOR, and PHO pathways [126]. Multi-omics integration to develop network maps, such as the maize comprehensive network map across genomic, transcriptomic, translatomic, and proteomic levels, illustrates the power of network-based approaches to identify functional gene modules and regulatory pathways relevant to the transport of nutrients [168].

### 8.2. Mathematical Models of Hyphal pi Uptake and Depletion Zones

Mathematical modelling has been an essential tool for quantifying the effect of extraradical hyphae on Pi plant uptake and improving the understanding of the spatiotemporal dynamics of phosphorus depletion zones within the soil. Schnepf and Roose [169] developed the first quantitative mathematical model describing the relationship between solute uptake by fungal mycelia and uptake by roots. This model showed that hyphal uptake is the dominant mechanism of phosphorus removal from soil and also that the depletion zone created by a single hypha overlaps with other depletion zones within just a few hours. Furthermore, this modelling indicated that Pi transfer between the fungus and root is a critical rate-limiting step for phosphorus acquisition during the mycelial phase [170]. Based on this research, Schnepf et al. [171] continued their modelling work to determine how the various growth and uptake patterns of AM fungi influence the soil depletion of phosphorus and the overall influx of phosphorus into mycorrhizal roots. The modelling showed that when all external hyphae contribute to Pi uptake, the fungus is the main source of phosphorus; when uptake is limited only to hyphal tips, the fungus is not as dominant of a supplier of phosphorus.

### 8.3. Databases and Computational Resources

In tandem with the accumulation of mycorrhizal genomic data and phosphorus cycling field data, specialized databases and computational tools have also been developed (Table 3). The PCycDB database provides a comprehensive and accurate repository of phosphorus cycling genes which enable the rapid metagenomic profiling of phosphorus transformation pathways in soil microbial communities including mycorrhizal fungi [172]. Genome sequencing and annotation of AM fungi have generated critical resources to understand the unique genomic organization, regulation, and evolutionary history of these obligate symbionts [173]. These genomic resources serve as foundation in the reconstruction of metabolic networks and the identification of candidate Pi transporters involved in the still-elusive step of fungal Pi efflux into the symbiotic interface [7]. Thus, computational tools for network visualization, pathway enrichment analysis, and genome-scale metabolic reconstruction continue to expand the bioinformatics toolkit available to the mycorrhizal research community.

### 8.4. AI and ML in Prediction of Phosphorus Abundance

ML and AI provide new predictive models for phosphorus cycling along the soil–mycorrhizae–plant continuum. An analysis of soil nutrient data, including phosphorus, using random forest and extreme gradient boosting models predicted crop yield with a reported accuracy of 86% [174]. Use of support vector machine regression and near-infrared spectra allowed for rapid predictions of total soil available phosphorus. Additionally, using portable X-ray fluorescence specifications and random forest models has enabled the determining of soil fertility parameter measurements (including phosphorus) for tropical soils [175]. Using deep learning neural networks and feature fusion modelling enhance existing fertilizer recommendations systems by capturing complex trends in soil nutrient datasets [176]. Although the results at the soil scale represent significant progress in predicting soil-related conditions, there are additional benefits to using physics-based models in mathematically simulating or replicating phosphate depletion areas around AM fungal hyphae and plant roots [169]. Through the modelling of simulated data, external hyphae have been shown to provide the majority of phosphorus needed for plant absorption relative to other forms of phosphorus uptake [169]. Future studies will develop methods for predicting the efficiency of phosphorus delivered to the root–fungal interface area relative to hyphal tip density and the pattern of anastomosis between neighbouring hyphae [171]. By integrating hyperspectral, soil, and fungal colonization data with the digital phenotyping of plant AI applications, predictions of phosphorus translocated from soil to the cytoplasm of plants have been developed [177]. Use of deep learning to model soil spectral data has produced estimates for several soil parameters, including estimates for the amount of phosphorus in each of the phosphorus pools of soil [178,179]. However, there is currently limited evidence of the ability of AI and mycorrhizal-specific transport models to discriminate between phosphorus delivered to plants using available datasets to validate models. For predictive capabilities to be realized, future studies should use ML algorithms linked with phthalate scale transport equations to predict phosphorus moving from the soil to the plant cell through fungal networks.

**Table 3:** Computational tools and databases for mycorrhizal phosphorus research.

Tool/Database	Application in Mycorrhizal P Research	URL	Key Functional Role	Reference
UNITE/PlutoF (PROTAX-fungi)	A web-based platform integrating the UNITE fungal ITS database with the PROTAX-fungi probabilistic classifier. Used for taxonomic placement of mycorrhizal fungal ITS sequences from environmental samples (roots, soil), enabling identification of AM fungi and ectomycorrhizal fungi involved in P uptake.	https://unite.ut.ee/ https://plutof.ut.ee accessed on 1 April 2026	Provides probabilistic taxonomic assignment of fungal ITS sequences with confidence scores, critical for profiling mycorrhizal communities in phosphorus-amended or phosphorus-limited soils.	[180]
MAFFT	A high-speed multiple sequence alignment tool based on fast Fourier transform. Essential for aligning PHT gene sequences, PHR transcription factors, or ribosomal markers from mycorrhizal fungi and host plants for phylogenetic analysis.	https://mafft.cbrc.jp/alignment/server/ accessed on 1 April 2026	Provides rapid and accurate multiple sequence alignment of phosphorus-related gene families across mycorrhizal fungi and plant species for evolutionary and functional studies.	[181,182]
SILVA rRNA Database	A comprehensive, quality-checked database of aligned small and large subunit rRNA sequences from all domains. Widely used for the phylogenetic placement of mycorrhizal fungal sequences in amplicon-based community studies examining phosphorus effects on fungal community composition.	https://www.arb-silva.de accessed on 1 April 2026	Serves as a reference for rRNA-based taxonomic assignment of fungi (including mycorrhizal) in amplicon sequencing studies investigating phosphorus-driven community shifts.	[183]
JGI MycoCosm/1000 Fungal Genomes	The DOE Joint Genome Institute portal provides access to hundreds of fungal genomes, including mycorrhizal species (ectomycorrhizal, arbuscular, ericoid); used for phosphorus transporter gene families and conducts comparative genomics of phosphorus acquisition strategies.	https://mycocosm.jgi.doe.gov accessed on 13 April 2026	Enables comparative genomics of phosphorus uptake and phosphorus metabolism pathways across diverse mycorrhizal fungi, revealing evolutionary adaptations for phosphorus acquisition.	[184]

## 9. Biological Engineering for Phosphorus Acquisition

Recent advances in biological engineering have opened new avenues for the enhancement of mycorrhizal phosphorus acquisition through three major strategies: (i) transgenic approaches targeting Pi transporters, (ii) engineering the PHR-SPX regulatory node, and (iii) broader strategies for the improvement in phosphorus acquisition efficiency.

### 9.1. Transgenic Approaches Targeting pi Transporters

The plant PHT1 family of Pi transporters plays a central role in both direct and mycorrhizal Pi uptake pathways [8,185]. These transporters are essential for Pi acquisition from the soil and its redistribution within the plant. Transgenic approaches have focused on overexpressing high-affinity Pi transporters to enhance uptake capacity. For instance, root epidermis-specific expression of the wheat phosphate transporter TaPT2 has been found to enhance plant growth significantly under both Pi-replete and Pi-depleted conditions [186]. Furthermore, understanding the mycorrhiza-specific Pi transport pathway whereby mycorrhizal-inducible PHT1 members (e.g., OsPT11 in rice, MtPT4 in *Medicago*) are expressed exclusively in arbuscule-containing cells has provided targets for genetic manipulation to boost symbiotic phosphorus transfer [7,8].

### 9.2. Engineering the PHR-SPX Regulatory Node

A breakthrough in the plant Pi signalling has been the elucidation of the PHR-SPX regulatory module [12]. Under Pi-sufficient conditions, SPX proteins (SPX1, SPX2, and SPX4) interact with PHR transcription factors to prevent their binding to PHR1-binding sequences (P1BS) in target gene promoters, and thus Pi-starvation responses are repressed [13,121]. Under Pi limitation, SPX proteins are degraded via the 26S proteasome pathway, releasing PHR transcription factors to activate the expression of Pi-starvation-induced genes [187]. Notably, this same regulatory module governs AM symbiosis. PHR transcription factors promote mycorrhizal colonization by the activation of AM-induced genes having P1BS motifs in their promoters, while SPX proteins suppress symbiosis under high Pi [12]. SPX4 has been shown to modulate both PHR1-dependent and PHR1-independent transcriptional responses in shoots which reveal additional layers of complexity in Pi signalling [188]. Engineering this regulatory node, for instance, by generation of SPX mutants that uncouple PHR activity from Pi sensing has a considerable potential to sustain AM symbiosis even under Pi-replete conditions, which will ultimately help maximize phosphorus acquisition efficiency.

### 9.3. Strategies for Improving P Acquisition Efficiency

Beyond the molecular engineering of individual genes, a multifaceted approach integrating genetic, agronomic, and biotechnological strategies is essential for improving phosphorus acquisition efficiency (PAE) in crops [185,189]. PAE encompasses external phosphorus efficiency which is the plant’s capacity to extract phosphorus from soil that can be enhanced through optimized root architecture, increased exudation of phosphatases and organic acids, and strengthened AM symbioses [189,190]. The integration of bioengineering approaches with precision P fertilization and management practices has been advocated to develop smart crop cultivars with high phosphorus utilization efficiency [191]. Critically, while improving phosphorus acquisition is necessary, enhancing phosphorus utilization efficiency in the internal remobilization and metabolic use of phosphorus may represent an even more powerful strategy for modern crops, as most crops already possess relatively efficient uptake capacity but suffer from poor phosphorus translocation and remobilization [192]. The integration of these engineering strategies with the growing information of mycorrhizal Pi transport mechanisms promises to deliver crops with enhanced productivity under limited phosphorus environment which reduces reliance on non-renewable phosphate fertilizers [121,190].

### 9.4. CRISPR/Cas9 Genome Editing

Recent advances in CRISPR/Cas9 genome editing have opened unprecedented opportunities to re-engineer this Pi transport circuitry for enhanced phosphorus acquisition efficiency [191,193]. With the application of CRISPR/Cas9 to target the promoter regions or coding sequences of specific PHT1 family members, it is possible to (i) disrupt negative regulators of mycorrhizal Pi transport that de-repress symbiotic Pi uptake; (ii) edit cis-regulatory elements of mycorrhiza-specific Pi transporter genes (e.g., PT11, PT13 in rice, PtPT10 in poplar) to boost their AM-inducible expression; (iii) modify Pi transporter protein domains to alter substrate affinity or localization [7]. The functional redundancy among PHT1 members necessitates the careful selection of editing targets, but knockout studies of key symbiotic transporters have proven non-redundant roles for certain isoforms [194]. For instance, silencing of rice PT11 disrupted AM symbiosis and reduced mycorrhizal Pi delivery by 70% [195], which confirm that these transporters are rate-limiting bottlenecks in the phosphorus express.

## 10. Conclusions and Future Prospects

We conclude that the journey of phosphorus from soil to cell exhibits a chain of tightly regulated transport events through multiple checkpoints. All checkpoints are well-coordinated by transporter proteins. The mycorrhizal phosphorus express is a highly regulated pathway that involves fungal Pi uptake from soil, translocation through hyphae, and release into the plant symplast which are operated under tight regulatory surveillance at multiple checkpoints. Through the synthesis of literature, we are in opinion that under Pi deficiency, PHR transcription factors activate mycorrhizal-associated Pi transporter genes, while under Pi sufficiency, SPX proteins inhibit PHR activity, and restrict fungal symbiosis. An example of this type of regulatory circuitry, which serves as a master checkpoint, linking the commitment to symbiosis with the needs of the plant for nutrients, is local to the periarbuscular interface, where specific mycorrhizal Pi transport proteins on the plant side serve both as conduits for phosphorus flux and as gatekeepers for the stability of the symbiosis by preventing arbuscular degeneration when they are not functioning properly.

In addition, there are many other areas that should be addressed in future research efforts. The first and most significant missing area in our knowledge is the identification and regulation of mycorrhizal Pi exporters that facilitate the unloading of phosphorus from the fungus into the apoplast of the interface. Future research should employ the heterologous expression of SYG1/XPR1 homologues in yeast *pho84* mutants as a functional validation system to determine their precise role in phosphate export across the mycorrhizal interface. Second, the interplay between the PHR-SPX module and symbiosis-specific transcription factors that coordinate nutrient exchange demands further extensive and critical investigation. Third, structural and biophysical studies of SPX-PHR complexes and their modulation by inositol pyrophosphates exhibit the mechanistic basis of Pi sensing. Fourth, exploitation of natural variation in mycorrhizal regulatory checkpoints across genome-wide association studies and genetic engineering will unlock the potential to upregulate symbiotic Pi delivery in crops. Fifth, a multidisciplinary approach which combines transcriptomics, functional analysis, structural biology, and host-induced gene silencing could help us resolve the issues related to identification and the well-functioning of fungal arbuscular transporters. Finally, the integration of mycorrhizal phosphorus management with sustainable agricultural practices such as reduced Pi fertilizer inputs and improved Pi-use efficiency represents a critical translational goal for sustainable food and environmental security.

## Figures and Tables

**Table 1:** Important PSB genera associated with AM hyphae, mechanisms of phosphorus solubilization.

PSB Genus/Species	Association With AM Fungi	Major Phosphorus Solubilization Mechanism	Supporting Evidence/Key Findings	Reference
*Rahnella aquatilis* HX2	Direct transport along extraradical AM fungal hyphae; bacteria moved as a cohort/biofilm on hyphal surface through a thick water film surrounding hyphae to discrete organic phosphorus patches	Enhanced organic phosphorus mineralisation in nutrient patches; transfer required hyphal exudates as energy source	AM fungal hyphae translocated PSB to organic phosphorus patches, thereby enhancing organic phosphorus mineralisation under both culture and soil conditions; without hyphal exudates, bacterial transfer to phosphorus patch did not occur	[39]
*Rahnella aquatilis* HX2.	Direct cooperation with AM hyphal exudate-mediated the interaction with *Rhizophagus irregularis*	Phosphatase-mediated mineralization of organic phosphorus (phytate), triggered by AM fungal—fructose signalling, leading to release of Pi	AM fungi-exuded fructose-stimulated bacterial phosphatase gene expression, enhanced protein secretion and phosphatase release, increased phytate mineralization, and consequently promoted phosphorus uptake by AM fungi; demonstrates fructose as both carbon source and signalling molecule	[62]
*Rahnella aquatilis* HX2	Develops near or on the surface of extraradical hyphae of *Rhizophagus irregularis* MUCL 43194; explicit hyphal surface interaction/hyphosphere dialogue	Focuses on metabolic regulation of bacterial TCA cycle, not direct phosphorus solubilization assay	AM fungi induced transcriptional changes in gltA, icd, and kgdhc, indicating regulation of bacterial ATP production and metabolism at the hyphal surface; supports a fine-tuned AMF–PSB molecular dialogue in the hyphosphere	[63]
*Rahnella aquatilis*	Direct hyphosphere interaction with hyphae of *Rhizophagus irregularis*	Phosphatase-mediated organic phosphorus mobilization (phytate) regulated by PhoR two-component system; gluconic acid production pathway transcriptionally inhibited	Presence of AM hyphae stimulated carbon-sensing and nutrient-sensing two-component system genes; PhoR two-component system enhanced phosphatase gene expression while repressing genes related to gluconic acid production; demonstrates two-component system-mediated sensing of hyphal carbon signals for efficient phosphorus mobilization in hyphosphere	[64]
*Streptomyces* spp., *Pantoea* spp., *Bacillus* spp.	Enhance AM fungi development and spore production; explicit association within tripartite plant–mycorrhiza helper bacteria–AM fungi system	Phosphate solubilization reported as a plant growth-promoting trait.	Genome mining revealed genes for siderophore production, aromatic compound degradation, and secondary metabolites; enhanced AM fungi growth and spore production, along with phosphate solubilization, ammonia production, and cellulolytic enzyme synthesis	[65]
*Pseudomonas* sp. strain F1G, *Pantoea* strain C1, *Micrococcus* strain F3,	AM fungi and plant growth-promoting bacteria inoculation in plant roots/pots	Phosphate solubilization	PGPB improved plant growth (tomato and corn) under rock phosphate; AM fungi improved growth mainly in tomato under triple superphosphate; plant growth-promoting bacteria effects linked to increased root length; AM fungi effects linked to improved mineral nutrient content	[66]
*Pseudomonas aeruginosa*, *Pseudomonas putida*, *Serratia plymuthica*	Interaction with *Glomus intraradices* external mycelium	Phosphate solubilization of sparingly soluble phosphorus sources associated with pH changes in the medium	AM fungal external mycelium and mycorrhizal roots enhanced phosphate solubilization in interaction with PSB beyond single cultures; effects were correlated with medium pH changes	[67]
*Bacillus megaterium*	Interaction occurs in alfalfa rhizosphere soil with AM fungi *Funneliformis mosseae*	Phosphate mobilization associated with alkaline phosphatase activity and organic acid production (malic acid, oxalic acid, acetic acid, total organic acids)	Double inoculation with AMF + *Bacillus* significantly increased soil alkaline phosphatase activity, organic acid content, soil organic matter, and improved phosphorus uptake efficiency; effects were strongest at 100 mg kg^−1^ P_2_O_5_ application rate	[68]

## Data Availability

Not applicable.
